# Prevalence of Rifampicin resistance tuberculosis among presumptive tuberculosis patients in Egypt-2021: a national health facility-based survey

**DOI:** 10.1186/s12879-023-08807-7

**Published:** 2024-02-15

**Authors:** Wagdy Amin, Mohsen Gadallah, Amal Salah, Mervat Rady

**Affiliations:** 1 Chest Diseases Department, Ministry of Health and Population, Cairo, Egypt; 2 Community, Environmental and Occupational medicine department, Faculty of Medicine-Ain, Shams University, Cairo, Egypt; 3 National TB Control Program (NTP), Cairo, Egypt

**Keywords:** Mycobacterium tuberculosis, Survey, Rifampicin resistance, Prevalence

## Abstract

**Background:**

The magnitude of MDR-TB cases was noticeable in Egypt. However, the last national survey was 11-years ago. The current survey was conducted to determine the prevalence of rifampicin resistance among sputum smear-positive pulmonary tuberculosis patients in Egypt.

**Methods:**

A national health facility-based cross-sectional study was conducted in 14 randomly selected governorates in Egypt between August 2020 and September 2021. All presumptive TB cases, either new or previously treated according to WHO definitions, with no gender, age, or nationality limitations, and provided informed consent were included in the study. Each patient completed a case report form (CRF). The CRF included socio-demographic and clinical data. Sputum samples were collected according to standard techniques and cultured on Lowenstein-Jensen (L-J) medium. Gene X-pert test was carried out first on the samples for simultaneous identification of MTB and rifampicin resistance. The prevalence of RR was calculated using crude, cluster, and weighted methods. Factors associated with RR were analyzed by bivariate and multivariate techniques.

**Results:**

Among the total 849 presumptive TB patients enrolled in the study, 710 (83.6%) patients were subjected to Gene X-pert testing (MTB/RIF). The crude prevalence of RR was 3.32% (95% CI: 1.89–4.76%) among the new cases and 9.46% (95% CI: 2.63–16.29%) among the retreated cases with an overall estimate of 3.99%; (95% CI: 2.51–5.47%). By cluster analysis the overall prevalence of RR was 5.01% (95% CI: 2.90–7.13). Factors associated with the prevalence of RR were co-morbidity with bronchial asthma, drug abuse and history of contact with a family member with TB.

**Conclusion:**

The prevalence of RR among either new or retreated cases TB patients was lower than the previous Egyptian rates in 2010–2012. The strongest predictor associated with RR was comorbidity with bronchial asthma.

## Background

Tuberculosis (TB) is a major cause of ill health and one of the leading causes of death worldwide [1]. Early, rapid and accurate diagnosis of all TB patients and prompt treatment with any form of drug-susceptible or drug-resistant TB is fundamental. Identifying the pattern of drug resistance will ensure that the most effective therapy can be selected [1, 2]. Drug resistance tuberculosis is a major public health problem that threatens progress made in reduction of the incidence and mortality rates of TB globally. Also, Drug-resistant TB is a major contributor to antimicrobial resistance worldwide. Deaths from drug resistance TB account for about one third of all anti-microbial deaths worldwide [3].

Multidrug-resistant (MDR) TB is defined by resistance to both rifampicin (RIF) and isoniazid (INH), the two core drugs used in the treatment of TB which requires an extended duration of treatment with less-effective drugs.

Resistance to RIF is considered a surrogate marker for MDR-TB. Extensive drug-resistant TB (XDR-TB) is defined as resistance to INH and RIF plus at least one fluoroquinolone (e.g., moxifloxacin) and one second-line injectable drug (e.g., kanamycin) [4, 5].

Globally in 2020, 71% (2.1/3.0 million) of people diagnosed with bacteriologically confirmed pulmonary TB were tested for rifampicin resistance. Among these, 132 222 cases of MDR/RR-TB and 25 681 cases of pre-XDR-TB or XDR-TB were detected, for a combined total of 157 903 cases. Drug resistance TB (DR TB) is difficult to diagnose as it requires specific laboratory tests not easily accessible to patients in particularly those living in low income and low-middle income countries. In addition, DR TB is harder to cure than those with susceptible TB, because the treatment requires two years versus 6 months for susceptible and some drugs are toxic and may lead to severe adverse events [1].

Egypt is considered as a TB low/middle burden country with an estimated incidence of 11 per 100,000 people per year. The magnitude of MDR-TB cases was noticeable in Egypt, especially among previously treated cases. The rates of MDR were studied in a number of national and governorate-based surveys. The first Egyptian drug resistance survey was conducted in 2002 with the detection of MDR-TB in 11.4% of the total TB cases (97out of 849), of which 2.2% were new cases and 38.2% were previously treated. The latest (second) survey in Egypt was between 2010 and 2012 and the results showed that the overall MDR-TB rate was increased to 3.4% among the TB new cases and decreased to 32.4% among the previously treated cases [6]. Since 2012, there was no national based survey for MD TB. However, there were few studies conducted at local level in some governorates to measure the prevalence of RR and factors associated with it. These studies are mainly retrospective based on records while few studies were cross-sectional or prospective [7–9].

Information about the current prevalence and patterns of MDR-TB at national level in Egypt is crucial in establishing effective TB therapeutic regimens. The objectives of this Egyptian national survey were to measure the prevalence of Rifampicin Resistance (RR), MDR-TB, and resistance to second line agents among newly and previously treated cases who, in response to direct questioning, reported having received one month or more of anti-TB drugs in the past, or there is documented evidence of having received one month or more of anti-TB drugs. The survey also aimed to identify the probable risk factors associated with the prevalence of RR among pulmonary TB patients. Identifying these factors will help the Ministry of Health and Population (MOHP) develop tailored prevention and control interventions targeting the determined high- risk groups.

## Methods

This research paper is a part of the national MDR-TB survey conducted in Egypt during the period from August 2020 to September 2021; titled “Tuberculosis Drug Resistance Survey-Egypt − 2021.”

### Study design

The survey was a national health facility-based cross-sectional study conducted following the WHO Guidelines for surveillance of drug resistance in tuberculosis [10]. The study population was all TB presumptive patients with clinical symptoms and signs suggestive of TB who visited the study selected TB diagnostic units during the survey period.

### Inclusion and exclusion criteria

A patient was eligible for inclusion in the survey if he/she was present as a presumptive TB case (new or previously treated patient), according to the WHO definitions [10]. Only consenting patients who could produce sufficient volumes of good-quality sputum were included. Patients were excluded if they declined to give informed consent/assent to participate in the survey, an extra-pulmonary case, new patients who had started anti-TB treatment for > one month (as their cultures might fail to grow), or retreated patients if they had already started retreatment regimen after being re-registered.

### Study overview

All consecutive presumptive pulmonary TB cases, which provided informed consent at the selected facilities during the survey period, and were positive by direct smear microscopy for TB bacilli were included in the survey. Each patient had a case report form (CRF) completed through a direct patient interview by a trained healthcare worker at the health facility and in addition had 2 spot survey-specific sputum samples collected. The CRF collected demographic, clinical, enrolment criteria, and risk factors information.

The CRF with the corresponding samples were sent to the peripheral (inside the diagnostic unit) or intermediate labs at the governorate where Gene X-pert analysis and liquid mycobacterial culture (if available) were done. Then, the sputum specimens or the cultures deposits were sent to the national central lab in Cairo for drug susceptibility testing against a panel of first-line and second-line anti-TB drugs on Mycobacterium tuberculosis-confirmed isolates, however, culture results were not included in this article.

### Sample size

Sample size was calculated based on new patients only as recommended by WHO [10]. Retreatment patients were sampled on convenience. The number of new patients required was determined using Stat Calc in Epi-Info version 6, and was based on the following criteria: (1) total number of TB new cases as reported from the official Ministry Of Health and Population (MOH-P) report = 3258, (2) the estimated proportion of MDR-TB by WHO-2018 country profile = 280/8448 = 3.3%, (3) absolute precision = 1.5%, (4) a confidence interval of 95%. To adjust for the cluster design effect, the calculated sample size was multiplied by 1.5 based on variations in clusters obtained from the previous national MDR survey in 2010. The calculated sample size was finally increased by 5% to account for expected loss of cases during the survey. Considering all the previous assumptions, a minimum of 737 new TB pulmonary patients were targeted for recruitment in addition to all the retreated eligible TB cases during the intended year-long study period. A total of 849 cases were included, including 769 new cases and 80 retreated cases.

### Sampling technique

The survey was conducted in 14 randomly selected governorates proportional to the patient load of sputum smear-positive TB cases reported to the National TB Control Program in 2020.

A weighted probability-proportional to size cluster sampling technique was used. With this design, the TB management units (TMUs) were randomly selected. A sampling frame was developed from the units’ official list which was distributed all over Egypt in the 27 governorates in 2018. Each cluster sample size was 25 (737/30). Among the participated governorates, 30 clusters were selected covering 63 TMUs. Quality of data was ensured through vigorous training of survey teams before the field work, cross-checking the original forms during support visits and during data monitoring missions all through the survey duration.

### Statistical analyses

Data were exported to SPSS version 22 (Chicago, Illinois, USA) for analysis. Categorical variables were summarized using frequencies and percentages. The statistical analysis was used to determine the crude RR estimates among TB cases. As the missing data in key variables were not exceeded 5%, data imputation was not performed. Probability weighting was applied, in order to address potential bias in enrolments. All risk factors associated with RR were analyzed using Chi squared (X^2^) or Fisher exact tests. Risk factors exhibited significant associations with RR in bivariate analyses were re-analyzed by multivariate logistic regression model. Level of significance at all analyses was considered at P- value < 0.05.

## Results

**Fig. 1:**
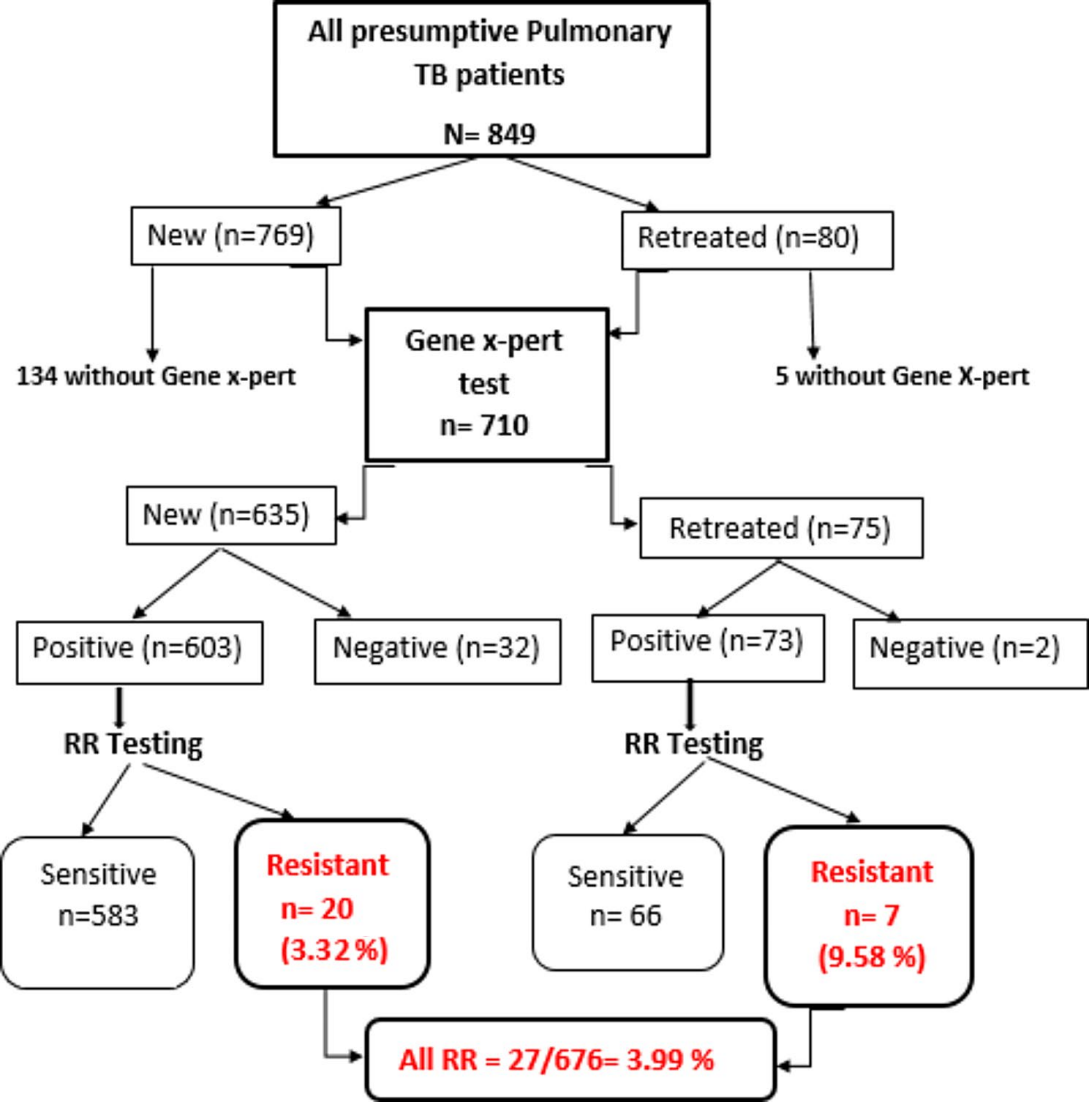
Flow Diagram of Gene X-pert results

A total of 849 presumptive pulmonary patients (769 new cases and 80 retreated cases) were screened and enrolled in the survey. Among the total 849 presumptive TB patients, 710 patients (635 new and 75 retreated) were subjected to Gene X-pert testing (MTB/RIF). The results showed that 676 patients (95.2%) were positive for MTB (603 new patients and 73 retreated patients). Of those 676 MTB patients, 27 (3.99%, 95% CI = 2.51–5.47%) patients were resistant to Rifampicin (RR); 20 (3.32%, 95%CI = 1.88–4.75%) out of the 603 new patients were RR while 7 (9.59%, 95%CI = 2.67–16.51%) out of the 73 retreated patients were RR (Fig. 1; Table 1).

In order to correct for the cluster design of this survey, the cluster prevalence of rifampicin resistance was recalculated taking into consideration the cluster level as the unit of analysis instead of individual-level data (Table 1). The overall cluster prevalence of RR was 5.01% (95% CI = 2.90–7.13%), while among new patients was 4.09% (95% CI = 2.18–5.99%), and for the retreated patients, was 8.48% (95% CI = 0.0–17.49%).

To overcome the differences in cluster size of the new cases which happened in the field and to get a more accurate result, we applied the weighting statistical method in calculating the prevalence of RR among new cases only. The weighted prevalence of RR among new cases was 3.84% (95% CI = 2.28–5.38%).

**Table 1:** Prevalence (%) of Gene X-pert Rifampicin Resistance (RR) among TB positive cases

Method of Calculation	Prevalence of RR (95% CI)		
	All TB patients N = 676	New Patients N = 603	Retreated Patients N = 73
Crude analysis	3.99 (2.51–5.47)	3.32 (1.88– 4.75)	9.59 (2.67–16.51)
Cluster analysis*	5.01 (2.90–7.13)	4.09 (2.18–5.99)	8.48 (0.0–17.49)
Weighted analysis		3.84 (2.28–5.38)	

* The prevalence and 95%CI were calculated according to clusters

As seen in Table 2, the age of the study population ranged between 5 and 85 years and 56.5% were in the age group 21–40 years. The majorities of study participants were males (74.4%), ever married (61.2%), live in urban areas (71.6%), literate (64.2%), not working or has no official work (76.0%) and Egyptian (89.9%). Family history of the presence of TB patient was reported by 17.3%. As regard patients’ habits, 31.4% were current smokers, 5.2% were alcoholics, while 5.2% reported illicit drug abuse. Past history of diabetes and liver diseases were reported by 16.9% and 2.8% of the study population respectively, while bronchial asthma was reported by 9.9%. HIV was tested positive in 4 cases (0.6%).

Table 3 displays the factors associated with the prevalence of RR among the study TB patients. In bivariate analyses, the prevalence of RR showed slight variations between different age groups being highest among patients aged more than 60 years (6.6%). However, there were no significant differences between age groups (P = 0.370). Almost the prevalence of RR was equal between males and females. Also, the results of this study showed that educational level, residence, nationality and working status were not found to be associated with the prevalence of RR. Ever married patients experienced a significant higher prevalence (5.3%) of RR than single patients (1.9%). Patients with positive family history of TB were more prone to have higher prevalence of RR (8.5%) than those without history (2.9%), this difference was statistically significant (p = 0.005).

Regarding special habits and its association with the prevalence of RR, the results showed that only patients gave history of illicit drug abuse have a greater prevalence of RR (10.5%) compared with non-drug abusers (3.4%), this difference was found to be significant (p = 0.008). Both tobacco smoking and alcoholism were not associated with the prevalence of RR.

The survey results showed that only TB patients with bronchial asthma experienced greater prevalence of RR (p < 0.001). While patients with history of liver diseases or diabetes showed no differences in the prevalence of RR. None of the only four HIV cases detected by the survey were positive for rifampicin resistance. There was a significant difference in the prevalence of RR between new and retreated TB patients (Table 3).

In order to adjust for confounding variables those affect the prevalence of RR, a multivariate logistic regression model was applied and included all variables that showed significant association with RR (Table 4). The model included the following variables; marital status, history of family TB, co-morbidity with bronchial asthma, type of TB patient and drug abuse. The model retained only 3 variables that significantly predict the prevalence of RR. The strongest associated variable was co-morbidity with bronchial asthma with adjusted odds ratio of 38.24 (95%CI: 14.59–100.24) then came both positive family history with TB (adjusted odds ratio of 3.34; 95% CI: 1.24–9.02) and illicit drug abuse (adjusted odds ratio of 3.38; 95% CI: 1.01–11.30).

**Table 2:** Patients’ characteristics, special habits and comorbidities (n = 676)

Variables	Codes	Frequency	%
Age groups/years	< 20	64	9.5
	20–40	382	56.5
	41–60	169	25.0
	> 60	61	9.0
Sex	Males	503	74.4
	Females	173	25.6
Residence	Urban	484	71.6
	Rural	192	28.4
Marital status	Ever Married	414	61.2
	Single	262	38.8
Educational Level	Illiterate + Read &Write	242	35.8
	Literate	434	64.2
Nationality	Egyptian	608	89.9
	Others	68	10.1
Working Status	Has current work	162	24.0
	No work/No official Work	514	76.0
Family History of TB	No	546	80.8
	Yes	117	17.3
	Don’t know	13	1.9
Tobacco smoking	Non-Smoker	256	37.9
	Current Smoker	212	31.4
	Ex-Smoker	208	30.8
Alcohol	No	641	94.8
	Yes	35	5.2
Drug abuse	No	619	91.6
	Yes	57	8.4
Diabetes	No	562	83.1
	Yes	114	16.9
Bronchial Asthma	No	609	90.1
	Yes	67	9.9
Liver diseases*	No	650	97.2
	Yes	19	2.8
HIV **	No	659	97.5
	Yes	4	0.6
Type of Patient	New	603	89.2
	Retreated	73	10.8

*7 cases with missing data, ** 13 patients were not reported

**Table 3:** The association between the studied risk factors and Rifampicin Resistance (RR)

Variables	Codes	Count	RR (%)	X^2^ test, (p value)
Age groups/years	< 20	64	3 (4.7%)	3.144 P value = 0.370
	20–40	382	11 (2.9%)	
	41–60	169	9 (5.3%)	
	> 60	61	4 (6.6%)	
Sex	Males	503	20 (4.0%)	0.002 P value = 0.968
	Females	173	7 (4.0%)	
Residence	Urban	484	16 (3.3%)	2.105 P value = 0.147
	Rural	192	11 (5.7%)	
Marital status	Ever Married	414	22 (5.3%)	4.853 P value = 0.028
	Single	262	5 (1.9%)	
Educational Level	Illiterate + Read &Write	242	14 (3.2%)	1.866 P value = 0.172
	Literate	434	13 (5.4%)	
Nationality	Egyptian	608	26 (4.3%)	1.256 P value = 0.262
	Others	68	1 (1.5%)	
Working Status	Has work	162	4 (2.5%)	1.292 P value = 0.265
	No work/No official Work	514	23 (4.5%)	
Family History of TB	No	546	16 (2.9%)	8.067 P value = 0.005
	Yes	117	10 (8.5%)	
Tobacco smoking	Non-Smoker	256	11 (4.3%)	0.310 P value = 0.856
	Current Smoker	212	9 (4.2%)	
	Ex-Smoker	208	7 (3.4%)	
Alcohol	No	641	24 (3.7%)	2.017 P value = 0.156
	Yes	35	3 (8.6%)	
Drug abuse	No	619	21 (3.4%)	6.927 P value = 0.008
	Yes	57	6 (10.5%)	
Diabetes	No	562	19 (3.4%)	3.269 P value = 0.071
	Yes	114	8 (7.0%)	
Bronchial Asthma	No	609	8 (1.3%)	115.131 P value < 0.001
	Yes	67	19 (28.4%)	
Liver diseases*	No	650	26 (4.0%)	0.076 P value = 0.783
	Yes	19	1 (5.3%)	
HIV**	No	659	27 (4.1%)	P value = 1.00 (Fisher’s Exact)
	Yes	4	0 (0.0%)	
Type of TB patient	New	603	20 (3.3%)	6.681 P = 0.010
	Retreated	73	7 (9.6%)	

* 7 cases with missing data, ** 13 patients were not reported

**Table 4:** Predictors of RR using the Logistic regression analysis

Variables	Prevalence of RR (%)	Crude OR (95%CI)		Adjusted OR (95%CI)		
		cOR*	95% CI	P value	aOR**	95% CI
Marital status Single Ever married	1.9 5.3	1(Ref) 2.89	----- ---- 1.08–7.72	0.096	1 (Ref) 2.58	----- ------ 0.84–7.89
Family History of TB No Yes	2.9 8.5	1(Ref) 3.10	---- ---- 1.37–7.01	0.017	1(Ref) 3.34	----- ----- 1.24–9.02
Drug abuse No Yes	3.4 10.5	1(Ref) 3.35	----- ---- 1.29–8.67	0.048	1(Ref) 3.38	----- ------ 1.01–11.30
Bronchitis Asthma No Yes	1.3 28.4	1(Ref) 29.74	------ ------ 12.37–71.46	< 0.001	1(Ref) 38.24	----- ------- 14.59 − 100.24
Type of TB patient New Retreated	3.3 9.6	1(Ref) 3.09	----- ------ 1.26–7.59	0.177	1 (Ref) 2.21	----- ----- 0.70–6.95

* cOR = Crude OR, ** aOR = Adjusted OR

## Discussion

Among the total 849 presumptive TB patients enrolled in the study, 710 (83.6%) patients (635 new and 75 retreated) were subjected to Gene X-pert testing (MTB/RIF). The coverage of Gene X-pert testing was 82.5% % for new cases and 93.7% for previously treated TB patients. These figures are higher than the global figures in 2020. According to WHO report, globally in 2020, 71% of patients with bacteriologically confirmed TB were tested for RR, up from 61% to 2019 [1]. Coverage of testing for rifampicin resistance world widely was 59% for new and 81% for previously treated TB patients. This reflects that Egypt is among the countries in which Gene X-pert testing becomes established well inside the TMUs.

Regarding RR using Gene X-pert testing, this survey showed that the prevalence of RR was 3.99% among the TB confirmed cases, a rate which is lower than the rate reported for any Rifampicin resistance from the previous national survey in 2010–2012; (12.8%). Moreover, the current rate is lower than the corresponding rates reported by previous Egyptian studies e.g. Farghaly et al. [7] (10.2%), Al Olimey et al. [8] (10.9%), Ibrahem and El-Helbawy [9] (12.0%). These studies were governorate-based with small sample size and not a national one which may explain the differences in the prevalence of RR. Also, the survey results were lower than the prevalence reported in India e.g. by Gautam et al. [11] (26.1%) and Mondal et al. [12] (8.91%). In addition, other countries in Asia, showed higher prevalence of RR as in Pakistan [13] (10.2%), Philippines [14] (19.3%), China [15] (5.9%). The difference between our rate and the previous rates may be due to that India and China are countries with high TB burden while Egypt is one of the low/middle TB burden countries. Comparison with African studies, our rate was more or less similar to the survey in South Africa [16] (4.6%), in Zimbabwe [17] (5.2%), and in Botswana [18] (5.4%). Other African countries reported higher prevalence of RR than our rate; in Ethiopia, Arega et al. [19] reported (9.9%); in Nigeria, Ulasi et al. [20] reported (6.8%) and in Eastern Democratic Republic of Congo, Bulabula et al. [21] reported (11%). The high prevalence of RR was observed in Eastern Europe and central Asia countries especially in Russia, Belarus, Moldova, Turkmenistan, and Tajikistan. These countries reported very high prevalence of RR among new cases that exceeds 20% [22].

Misdiagnosed, undiagnosed, or untreated drug-resistant TB contributes to sustained high prevalence of drug-resistant TB and high proportions of infectious drug-resistant TB cases among the community. Active drug-resistant TB is either primary resistance (a person has been infected with a drug resistant TB strain) or secondary resistance (result from inadequate or poor treatment quality), both are interconnected and have many reported contributing factors as inappropriate treatment guidelines, poor adherence to the treatment, unavailability of treatment, co-morbidity with HIV & Diabetes and substance abuse [23].

Regarding factors associated with high prevalence of RR, the current study showed that Bronchial Asthma (BA) is a strong predictor for RR-TB. Few studies reported the association between BA and MDR TB. Some studies as the one reported by Sambas et al. [24] in Saudi Arabia which used the term lung diseases, found that lung disease is a significant predictor for MDR TB. However, another study conducted in Somalia by Guled et al. [25] showed that BA was not associated with the prevalence of RR. Other chest diseases as Chronic Obstructive Lung Diseases (COPD) has been discussed as a potential risk factor for MDR TB. A systemic review and meta-analysis by Pradipta et al. [26], reported that patients with COPD are significantly more likely to have MDR-TB than non-COPD patients. Pulmonary comorbidity can promote the development of TB and its resistance to drugs by damaging innate lung defense, impairing lung function and changing lung structure. Another explanation was mentioned by Kiran et al. [27], who reported that CD4 + T lymphocytes were depleted in TB patients complicated with COPD and that patients with low CD3 and CD4 cell counts were susceptible to developing MDR-TB due to impaired IFN-gamma and IL-2 responses with lymphopenia. Also, it is possible that patients with COPD used many antibiotics which may render them more prone to MDR-TB.

Similar to our survey, history of contact with a family member with TB was strongly associated with rifampicin resistance (RR), MDR-TB and XDR-TB in an Indian study [28] and to resistance to more than one drug in Brazil [29]. Sharing the same socioeconomic level, overcrowding, air pollution and exposure with MDR infectious source may explain this relation [30].

The current study reported that illicit drug abuse is a risk factor of RR-TB (in both bivariate and multivariate analyses). There is no consensus about this association in the literature. Few studies reported positive association between intravenous drug and DR TB [31, 32]. In addition, one study reported association between illicit drug abuse and Isoniazid mono-resistance while not significant with Rifampicin mono-resistance [33]. Intravenous drug users and or drug abusers are more prone to deleterious effect on the immune system, non-adherence to treatment and take longer time to be converted to negative culture [32, 34, 35].

In the present study, RR-TB was significantly higher (bivariate analysis) among previously treated patients compared to the new cases. However, this association disappeared in the multivariate model. Various studies worldwide have established that previous treatment with anti-tuberculosis therapy is a major risk factor for RR-TB resistance [11, 13, 15, 17–19, 24–26]. This may be explained by poor compliance, lack of supervision, weak TB control program with interrupted drug supply, inadequate TB treatment, incorrect medical prescriptions, and unsatisfactory compliance by TB patients and physicians, as reported in the literature [13, 18, 24, 26, 36].

### Survey strengths and limitations

This survey was a national-based and its methodology followed the guidelines of WHO for conducting the National Survey for MDR. It is also performed during the tough times of COVID-19 lockdown. However, the survey had certain limitations, as our finding of the MDR-TB is an estimate and may not be generalizable to the whole community as culture and sensitivity were performed for a subset of patients (around one third) due to limitation in the resources and time constrain during the pandemic of COVID-19. This should be considered in interpreting our findings from the current survey. The cross-sectional nature of the current study design has not also established a causal relationship between the reported significant risk factors and RR-TB.

## Conclusion

Out of the 676 positive TB patients, 3.99% were resistant to Rifampicin (RR) using the gene X-pert testing. This rate is quite lower than the prevalence of RR (any) reported in the previous national survey in 2010–2012. The factors associated with high prevalence of RR were comorbidity with bronchial asthma, positive history of contact with a family member with TB, and illicit drug abuse. Periodic national survey is suggested to monitor the trend of MDR TB in Egypt and to identify the major contributor factors and manage them properly.

## Data Availability

The data sets are not publicly available. However, the datasets used during the survey are available on reasonable request and with permission from MOHP-Egypt.

## Abbreviations

TB: Tuberculosis
RR: Rifampicin Resistance
DR: Drug Resistance
MDR: Multidrug-resistant
TMUs: TB management units
XDR: Extensively drug-resistant
WHO: World Health Organization
CRF: Case report form
RIF: Rifampicin; INH:Isoniazid
CI: Confidence Interval
